# High-Throughput Sequencing Identifies 3 Novel Susceptibility Genes for Hereditary Melanoma

**DOI:** 10.3390/genes11040403

**Published:** 2020-04-08

**Authors:** Catarina Campos, Sofia Fragoso, Rafael Luís, Filipe Pinto, Cheila Brito, Susana Esteves, Margarida Pataco, Sidónia Santos, Patrícia Machado, João B. Vicente, Joaninha Costa Rosa, Branca M. Cavaco, Cecília Moura, Marta Pojo

**Affiliations:** 1 Unidade de Investigação em Patobiologia Molecular (UIPM) do Instituto Português de Oncologia de Lisboa Francisco Gentil E.P.E., Rua Prof. Lima Basto, 1099-023 Lisboa, Portugal; 2 i3S-Institute for Research and Innovation in Health, University of Porto, Rua Alfredo Allen 208, 4200-135 Porto, Portugal; 3 IPATIMUP—Institute of Molecular Pathology and Immunology, University of Porto, Rua Dr. Roberto Frias s/n, 4200-465 Porto, Portugal; 4 Unidade de Investigação Clínica (UIC) do Instituto Português de Oncologia de Lisboa Francisco Gentil E.P.E., Rua Prof. Lima Basto, 1099-023 Lisboa, Portugal; 5 Instituto de Tecnologia Química e Biológica António Xavier, Universidade Nova de Lisboa, Av. da República (EAN), 2780-157 Oeiras, Portugal; 6 Serviço de Anatomia Patológica do Instituto Português de Oncologia de Lisboa Francisco Gentil E.P.E., Rua Prof. Lima Basto, 1099-023 Lisboa, Portugal; 7 NOVA Medical School|Faculdade de Ciências Médicas, Universidade NOVA de Lisboa, Campo dos Mártires da Pátria, 130, 1169-056 Lisboa, Portugal; 8 Clínica de Risco Familiar do Instituto Português de Oncologia de Lisboa Francisco Gentil E.P.E., Rua Prof. Lima Basto, 1099-023 Lisboa, Portugal; 9 Serviço de Dermatologia do Instituto Português de Oncologia de Lisboa Francisco Gentil E.P.E., Rua Prof. Lima Basto, 1099-023 Lisboa, Portugal

**Keywords:** cutaneous melanoma, germline mutations, WES, hereditary melanoma

## Abstract

Cutaneous melanoma is one of the most aggressive human cancers due to its high invasiveness. Germline mutations in high-risk melanoma susceptibility genes have been associated with development hereditary melanoma; however, most genetic culprits remain elusive. To unravel novel susceptibility genes for hereditary melanoma, we performed whole exome sequencing (WES) on eight patients with multiple primary melanomas, high number of nevi, and negative for high and intermediate-risk germline mutations. Thirteen new potentially pathogenic variants were identified after bioinformatics analysis and validation. *CDH23*, *ARHGEF40,* and *BRD9* were identified as the most promising susceptibility genes in hereditary melanoma. *In silico* analysis of CDH23 and ARHGEF40 variants provided clues for altered protein structure and function associated with the identified mutations. Then, we also evaluated the clinical value of *CDH23*, *ARHGEF40,* and *BRD9* expression in sporadic melanoma by using the TCGA dataset (*n* = 461). No differences were observed in *BRD9* expression between melanoma and normal skin samples, nor with melanoma stage, whereas *ARHGEF40* was found overexpressed, and *CDH23* was downregulated and its loss was associated with worse survival. Altogether, these results reveal three novel genes with clinical relevance in hereditary and sporadic melanoma.

## 1. Introduction

Skin cancer incidence has been rising alarmingly fast, becoming a concerning public health issue [1]. Of all skin cancers, melanoma stands out due to its high invasive capacity, being considered one of the most aggressive skin cancers, accounting for ~69% of deaths caused by cutaneous malignancies [2]. True features of hereditary melanoma such as unilateral lineage, early onset of disease, and multiple primary lesions are quite rare, even in melanoma patients that report a family history of this neoplasm [3]. Family history comprises approximately 5–15% of melanoma cases; however, this does not imply that a single genetic mutation is being transmitted [4]; shared sun exposure and other risk factors are more plausible causes of melanoma among families with susceptible skin types [3]. Patients diagnosed with a single primary melanoma are at an increased risk of developing subsequent primary melanomas, which most likely occur within two years after the first diagnosis [5]. In fact, this has been demonstrated for 70% of melanoma patients who developed a second primary melanoma, showing the importance of close skin surveillance [6].

Non-melanoma skin cancer, other cancer types, gender, race, a higher number of nevi (especially dysplastic nevi), actinic skin damage, and family history of melanoma are all risk factors for hereditary melanoma [5,7]. Furthermore, hereditary melanoma has been associated with germline mutations in high-risk melanoma susceptibility genes (*CDKN2A, CDK4, TERT, POT1*) [8,9,10,11,12,13], polymorphisms in intermediate-risk melanoma susceptibility genes (*BAP1, ACD, TERF2IP, MC1R* and *MITF*) [14,15,16], and germline missense substitutions in *MITF* [17]. Germline mutations in *CDKN2A* are present in ~20–40% of multiple primary melanomas (MPM) cases [16]. *CDK4*, *POT1*, *ACD*, *TERF2IP,* and *MITF* germline mutations were identified in ~0.6–14.3% of MPM cases [16,18,19], while *MC1R* polymorphisms were detected in 60.5–82.1% [20,21] of MPM patients. Furthermore, despite being described in only two families, *TERT* mutations have been described as high-risk melanoma susceptibility genes. Regardless of the huge effort to identify novel susceptibility genes in melanoma patients, most hereditary melanoma genetic causes remain unknown.

In this work, we employed whole exome sequencing (WES) to identify novel susceptibility genes for hereditary melanoma in patients with MPM, who were negative for germline mutations in the genes *CDKN2A*, *CDK4,* and *MITF* (p.E318K). The novel identified genes were further assessed for their clinical potential using The Cancer Genome Atlas (TCGA) database that comprises of 461 melanoma cases and was analysed in silico. To our knowledge, this is the first study using WES to identify novel MPM susceptibility genes.

## 2. Materials and Methods

### 2.1. Institutional Approval

This work involving melanoma patients as well as healthy controls samples was approved by the Ethics Committee of Instituto Português de Oncologia Francisco Gentil de Lisboa (IPOLFG, UIC/829). Informed consent was obtained from all subjects (patients and healthy controls); all subjects were over 18 years of age. The methods were performed in accordance with good clinical practices guidelines and Portuguese law.

### 2.2. Biological Samples

DNA samples from patients diagnosed with MPM (*n* = 26), indexes with familial melanoma (*n* = 37), and healthy controls (*n* = 100−300; male to female ratio 1:1, at advanced ages (mean = 64 years old), were used.

The indexes with familial melanoma and patients diagnosed with MPM were obtained by the Unidade de Investigação em Patobiologia Molecular (UIPM) from Instituto Português de Oncologia de Lisboa Francisco Gentil (IPOLFG). All patients were monitored by the Department of Dermatology and Familial Risk Clinic from IPOLFG and were subjected to genetic testing according to criteria used in Portugal [22].

The healthy control samples were cancer and disease-free and obtained from Biobanco-iMM, Lisbon Academic Medical Center, Lisbon, Portugal.

All participants of this study were Portuguese and of Caucasian descent.

### 2.3. DNA Extraction

DNA of the family members was extracted from leukocytes using commercial kits (EZ1 DNA Blood kit) according to the manufacturer’s instructions (Qiagen, Hilden, Germany). DNA amount was quantified with a Qubit™ Fluorometer (Thermofisher, Grand Island, New York, USA).

### 2.4. Whole Exome Sequencing

Eight patients with MPM, of whom two were siblings, were selected for whole exome sequencing (WES) analysis performed by SourceBioScience NGS Service (Nottingham, UK). These patients were selected due to the fact that they were the most representative from our population, with a higher number of primary melanomas, and had been waiting for the longest period for a decision regarding their genetic condition. The patients had been diagnosed with MPM and previously screened for melanoma susceptibility genes *CDKN2A*, *CDK4,* and *MITF* (p.E318K) germline mutations. Genomic DNA libraries were enriched for exomic sequencing using Agilent SureSelect Human All Exon V6. The captured exonic sequences were sequenced using the Illumina NextSeq500 V2 (Illumina, Inc., San Diego, CA, USA) using one high-output flow cell at 75 bp paired-end reads. All reads were aligned to the Human Genome version GRCh38 (May 2017 version). The SRA accession number for sequencing data included in this study is PRJNA543971.

### 2.5. Variant Selection

**Figure 1:**
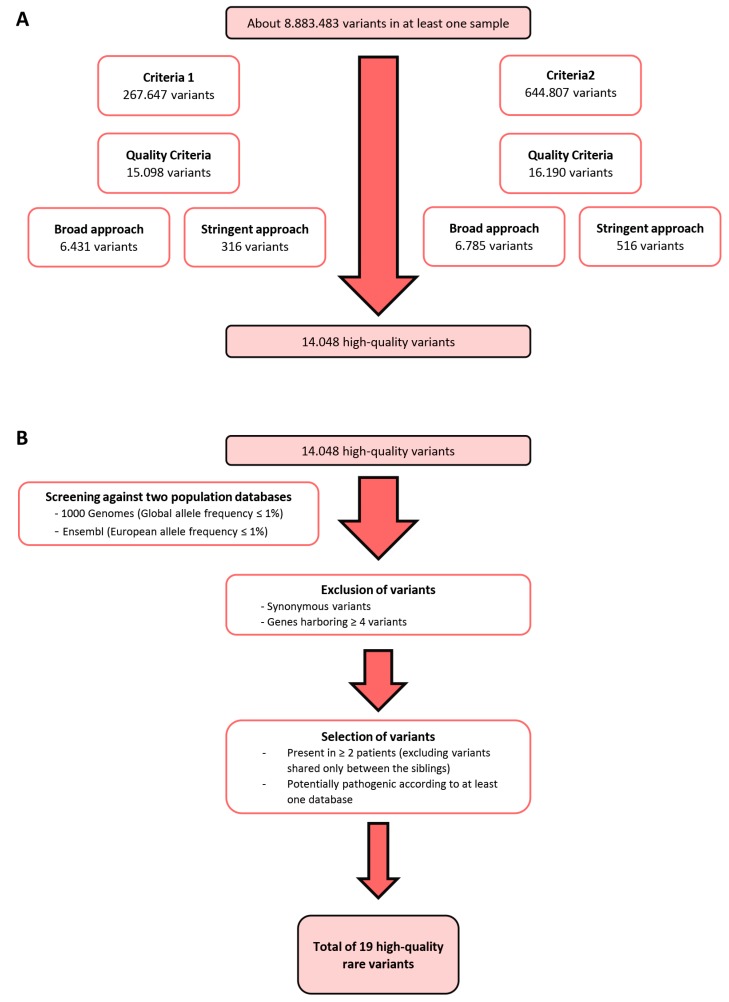
Variant selection. (**A**) Bioinformatics analysis was conducted using two criteria: Criteria 1 included potentially altered variants detected in two siblings and at least one other patient; Criteria 2 included potentially altered variants detected in at least two patients, excluding siblings. Only high-quality variants were included, and two approaches were used: A “broad approach” selecting only exonic variants and a “stringent approach” selecting exonic and intronic variants that had high quality, clinical significance, or predicted damaging effects. (**B**) Exclusion of variants with an allele frequency higher than 1% in the global and European populations, synonymous variants, and genes harbouring four or more variants, followed by selection of variants present in more than two patients and potentially pathogenic according to at least one database.

Bioinformatics analysis performed by Bioinf2Bio (Porto, Portugal) was first carried out as follows: Criteria 1, including potentially causative and altered genes detected simultaneously in the samples from the two siblings and in at least one other patient; Criteria 2, including potentially causative and altered genes detected in at least two non-sibling samples. Only variants with high-quality genotype, alignment, and a defined genotype were selected. These high-quality variants were then annotated to all protein-coding transcripts in the human genome. Two parallel approaches for variant selection covering each criterion were undertaken: A “broad” approach, consisting of all high-quality exonic variants; and a “stringent” approach, comprising all high-quality exonic and intronic variants that featured at least one of the following aspects: high quality predicted by Ensembl; clinical significance according to ClinVar, examined by Ensembl; predicted damaging effects by SIFT, Polyphen, MetaSVM or MetaLR. (Figure 1A). Common polymorphisms (allele frequency ≥ 1%) were excluded by additional criteria (Figure 1B). Complete description is provided in Supplementary Materials. WES data and patient clinicopathological data are aggregated in Table S5.

### 2.6. In Silico Analysis

In silico mutation analysis was performed for prediction of potentially deleterious effects of validated variants, using several tools: SIFT, Polyphen-2, Provean, MutationTaster, and FATHMM. Splice site prediction was calculated using Human Splicing Finder. In order to understand the possible molecular consequences of the identified *CDH23*, *ARHGEF40,* and *BRD9* mutations in the absence of structural data for each of the corresponding protein variants, structural models were generated using the SWISS-MODEL online server [23] (protocol detailed in Supplementary Materials). The models were inspected in Pymol [24] and the respective amino acid substitutions generated using the mutagenesis tool.

### 2.7. Expression and Prognostic Analyses of CDH23, ARHGEF40, and BRD9

The total number of cases analysed for mutation frequency are 994 cutaneous melanomas (TCGA = 448, Snyder = 64, Broad 2014 = 78, Broad 2012 = 121, Broad/DFCI = 26, Van Allen = 110 and Yale = 147). The total number of cases with survival data for each gene are BRAF = 419; CDH23 = 419; ARHGEF40 = 420; BRD9 = 421. Data was extracted from the cBIOPortal (http://www.cbioportal.org) [25,26].

*CDH23*, *ARHGEF40,* and *BRD9* gene expression data for cutaneous melanoma (SKCM) was extracted from Gene Expression Profiling Interactive Analysis (GEPIA) database (http://gepia.cancer-pku.cn) [27]. GEPIA database compromises RNA sequencing data from TCGA (SKCM n = 461, and normal n = 1) and normal GTEx (n = 557) samples. The Boxplots were generated to compare the expression levels between the tumour and normal skin samples. The violin plots were created based on the patient’s pathological stages. The expression data was transformed in log_2_ (transcripts per kilobase million + 1) and one-one-way ANOVA was used for differential analysis. The prognostic value was assessed using survival analysis from the GEPIA program. Patients were divided in low and high expression groups based on median expression cut-off for each gene. Overall survival and disease-free survival of SKCM patients were analysed using the Cox PH model.

### 2.8. Functional Enrichment Analysis

Co-expressed genes with *CDH23*, *ARHGEF40,* or *BRD9* were extracted from the SKCM TCGA dataset [25,26]. Only genes with Spearman > 0.5 and q value < 0.01 were considered positively correlated. Co-expressed genes with *CDH23*, *ARHGEF40,* or *BRD9* expression were then subjected to Gene Ontology (GO) and biological pathway enrichment analysis using PANTHER 14.0 (http://pantherdb.org) [28] against *Homo sapiens* background reference (GO database released 2018.12.19). The statistical over-representation was calculated using a binomial test and the results were considered significant at *p* < 0.05, after Bonferroni correction.

### 2.9. Genomic Mutation Analyses

To determine the frequency of *CDH23*, *ARHGEF40*, *BRD9*, *NRAS,* and *BRAF* mutations in SKCM samples, data recently re-annotated from TCGA at GDC (https://portal.gdc.cancer.gov) and cBioPortal was employed. The prognostic value of mutated genes was evaluated using the overall survival Kaplan-Meier tool from cBioPortal.

### 2.10. Variants Validation

The selected variants were validated by Sanger sequencing. First, polymerase chain reaction (PCR) was performed in a thermocycler (Biometra, Göttingen, Germany), utilizing 5 µL of AmpliTaq Gold™ 360 Master Mix (Applied Biosystems, Foster City, California, USA), 1–1,5 µL of forward and reverse primers (1 µM) (Invitrogen™) and 1–3 µL of DNA (20 ng/µL), comprising a final volume of 10 µL. The primer sequences used, along with their respective sequence length and optimal temperature of annealing are listed in Table S4. To confirm amplification of the fragments of interest, agarose gel electrophoresis was performed using an agarose gel at 2% (w/v), in TBE 1X (TBE Buffer 10×) (National diagnostics, Atlanta, Georgia, USA), to which 5% of ethidium bromide (0.5 µg/mL) (PanReac AppliChem, Darmstadt, Germany) were added. Electrophoresis was carried out on an ABI 3130 DNA analyzer (Applied Biosystems, Foster City, California, USA). Then, for unincorporated primer and non-amplified DNA degradation, 2 µL of a mix containing FastAP Thermosensitive Alkaline Phosphatase enzyme (1 U/μL) (Thermo Scientific, Grand Island, New York, USA) and Exonuclease I enzyme (20 U/μL) (Thermo Scientific, Grand Island, New York, NY, USA) in a proportion of 2:1, respectively, were added to each PCR product. The enzyme digestion reaction was performed in the same thermocycler aforementioned. The PCR sequencing reaction was performed using BigDye Terminator v1.1 sequencing kit (Applied Biosystems, Foster City, California, USA) according to the manufacturer’s instructions. Afterwards, samples were purified using a column-based DNA purification performed using the AutoSeqTM G-50 Dye Terminator Removal Kit (illustraTM, Brighton, UK).

Finally, samples were added to a 96-well plate (96-well PCR Microplates, AxygenTM) and sequenced using a 3130 Genetic Analyzer (Applied Biosystems, Foster City, California, USA) sequencer.

### 2.11. Statistical Analysis

In order to evaluate cumulative effects of the multiple variants in a genomic region identified in our analysis (*NTN4*, *MTCL1*, *FNDC1*, *CAND2*, *ITIH3*, *RPL32,* and *RNF213*), we conducted the following region-based aggregation tests: the burden test, which indicated if a region has a large proportion of causal variants with effects in the same direction; the sequence kernel association test (SKAT), which is more powerful in the presence of both risk-increasing and risk-decreasing variants or if there are many non-causal variants; and the omnibus test SKAT-O, which adaptively combines the SKAT and burden test statistics [29,30]. These analyses were conducted using R-package SKAT and all *p*-values were two-sided.

## 3. Results

### 3.1. Identification of Rare High-Risk Variants for Hereditary Melanoma

As per the filtering steps detailed in Figure 1A, 14,048 high-quality variants remained from WES data of eight patients with multiple primary melanomas (MPM), which were negative for all the susceptibility genes for melanoma (*CDKN2A*, *CDK4*, *MITF*, *BAP1*) and telomere maintenance complex genes such as Telomerase Reverse Transcriptase (*TERT*), Protection of Telomeres 1 (*POT1*), Shelterin Complex Subunit and Telomerase Recruitment Factor (*ACD*) and Telomeric repeat-binding factor 2-interacting protein 1 (*TERF2IP*). Additional criteria were applied (Figure 1B), leading to 19 rare high-quality variants, from which 13 were validated by Sanger sequencing (Table S1). To identify the most promising variants, we analysed their potential pathogenicity using several impact prediction servers (Table S2). In order to evaluate the pathogenicity of these 13 variants in MPM, we screened 18 additional MPM patients, and 37 patients with criteria for familial melanoma, 51 being negative and 4 positive for *CDKN2A* mutations (frequencies in Table 1).

Interestingly, when we screened these variants in the 37 indexes with familial melanoma a low variant frequency was observed, being absent in nearly half the cases (Table 1).

To confirm the rarity of the variants found and exclude specific polymorphisms of the Portuguese population, we assessed blood healthy controls. As shown in Table 1, most of the variants were polymorphisms (10 of 13), presenting a frequency >1% and <5%, with the exception for *MAP2K3* gene variant, which was a common variant with a frequency of 92%. Furthermore, the *BMX* and *CFAP47* variants were found in homozygosity in a healthy control, thus being excluded, as familial melanoma susceptibility is consistent with autosomal dominant inheritance. Importantly, we identified 3 rare variants in the *CDH23*, *ARHGEF40* and *BRD9* genes (0–0.7% frequency in healthy controls, Table 1) and confirmed their rarity using ExAC (0.007002, 0.001894 and 0.000464, respectively) and gnomAD (not found, 0.00186 and 0.000404, respectively).

In order to evaluate if these rare variants could synergize and increase melanoma susceptibility, we performed a region-based aggregation test analysis with all variants identified. We found a statistically significant cumulative effect of *NTN4*, *MTCL1*, *FNDC1*, *CAND2*, *ITIH3*, *RPL32* and *RNF213* variants in the MPM group, compared to the healthy control group (Table 2). Interestingly, *CAND2* and *RPL32* are in the same locus (3p25.2), strengthening the hypothesis that they could be co-segregated to the next generation and, consequently contribute together to increase MPM susceptibility.

We investigated *in silico* the possible molecular consequences of the identified *CDH23*, *ARHGEF40* and *BRD9* mutations by generating structural models, which were not obtained for BRD9.

Cadherin 23 is composed of an ectodomain comprising 27 extracellular cadherin (EC) repeats anchored to the cell membrane through a transmembrane helix, and a C-terminal cytosolic domain coupling CDH23 to the cytoskeleton [31]. Ca^2+^ binding at linker regions flanking each EC repeat is essential for the function of the tip-link, assembled by two cis homodimers of CDH23 and protocadherin-15 (PCDH15) connected tip-to-tip. Structural models of CDH23 EC4 (Table S3) were aligned in Pymol with the template yielding the highest score (PDB ID 5SZO), corresponding to EC repeats 1–4 of protocadherin γB7 (PCDHγB7; yellow ribbons in Figure S1, top and middle panels). Zooming-in on the Ala366Thr substitution (Figure S1, bottom panel), the mutated residue seems unlikely to affect protein folding, stability or aggregation, being located at the protein surface and replacing an uncharged side chain with a polar one. Therefore, such a pathogenic substitution could affect either the CDH23 homodimer assembly [31], or its interaction with other proteins. Protocadherin (PCDH) γB7 (PCDHγB7) contains several residues that are targets for post-translational modifications, particularly threonine mannosylation. Notably, PCDHγB7 EC3 residue Thr230 is mannosylated in crystal form 2 (PDB ID: 5SZP); this residue is perfectly aligned with the Thr366 from the CDH23 pathogenic variant herein studied (Figure S1). It is thus envisaged that the CDH23 p.Ala366Thr variant acquires an otherwise absent mannosylation site.

ARHGEF40, or Solo [32], a Rho-guanine nucleotide exchange factor (Rho-GEF), has posited roles in the maintenance of cell and tissue integrity against mechanical stresses [33]. Solo comprises a highly conserved N-terminal domain, a central region containing a CRAL/TRIO domain and spectrin repeats, and a C-terminal region containing a Dbl homology domain and a pleckstrin homology domain (DH-PH domain) [32]. The Arg834Cys substitution resulting from the herein identified pathogenic *ARHGEF40* mutation is located in the central region containing the spectrin repeats. Accordingly, the best models were generated against repeats 14–16 of β2-spectrin (Table S3, Figure S2), revealing that the substituted Arg834 is located in a disordered link between two α-helices, with its side chain within H-bonding distance (2.6–3.4 Å) with Glu858 (Figure S2; blue) or Gln851 (Figure S2; green) side chains. Substitution by a cysteine residue will likely result in the loss of these H-bonds, disturbing the conformation of this motif within the Solo central domain and eventually causing protein misfolding and/or aggregation.

### 3.2. The Impact of CDH23, ARHGEF40 and BRD9 in Sporadic Melanoma

**Figure 2:**
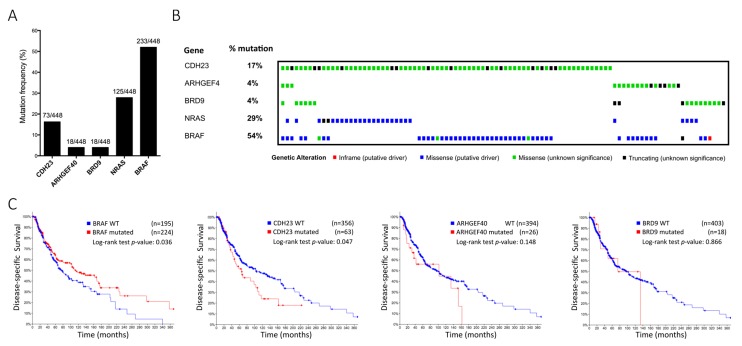
(**A**, **B**) Mutation frequency and heatmap from the TCGA dataset. Mutation significance is represented as follows: Inframe (putative driver)—red square; Missense (putative driver)—blue square; Missense (unknown significance)—green square; Truncating (unknown significance)—black square. (**C**) Disease-specific survival for *BRAF*, *CDH23*, *ARHGEF40,* and *BRD9* mutations in a combination of seven independent cohorts (TCGA, Snyder, Broad 2014, Broad 2012, Broad/DFCI, Van Allen and Yale). Total cases with survival data for each gene are *BRAF* = 419; *CDH23* = 419; *ARHGEF40* = 420; *BRD9* = 421.

We found that the novel identified variants and polymorphisms (Table 2) seemed to influence melanoma development. Particularly, we found that three rare variants in *CDH23*, *ARHGEF40* and *BRD9*, respectively, could be pathogenic on their own. Since the impact of these three genes in the context of sporadic melanoma remains to be fully elucidated, we further investigated their mutation frequency, along with frequent *BRAF* and *NRAS* mutations found in melanoma, in the TCGA database comprising a large cohort of melanoma patients (n = 448). As expected, *BRAF* and *NRAS* mutations were highly frequent (54% and 29%, respectively; Figure 2A). *CDH23* was also highly mutated (17%), missense and truncations being the predominant types of mutations. Many missense *CDH23* mutations were of unknown significance, demonstrating the importance of studying this gene and its alterations. *ARHGEF40* and *BRD9* were mutated in 18 of 448 melanoma patients (4%; Figure 2B). We also performed further mutation frequency analysis in 6 additional databases, which revealed the similar frequencies (Figure S3).

Since *NRAS* and *BRAF* mutations are mutually exclusive and *CDH23* mutations were detected simultaneously either with *NRAS* or *BRAF* mutations, we evaluated the prognostic value of these mutations. *BRAF* mutated samples had a significantly higher disease-specific survival (*p* = 0.036), as already described. Furthermore, data from patients with mutated *CDH23* revealed a lower disease-specific survival (*p* = 0.047). Contrarily, *ARHGEF40* and *BRD9* mutations did not reach a statistically significant prognostic value due to the small sample size of mutated cases (Figure 2C). Nevertheless, melanoma is characteristically a high mutational burden tumour type [34], and since gene length and mutation frequency are usually correlated, it is plausible that *CDH23* mutations do not constitute a proper marker of prognostic value and its high mutation frequency derives from large size of this gene (>419,000 bases).

**Figure 3:**
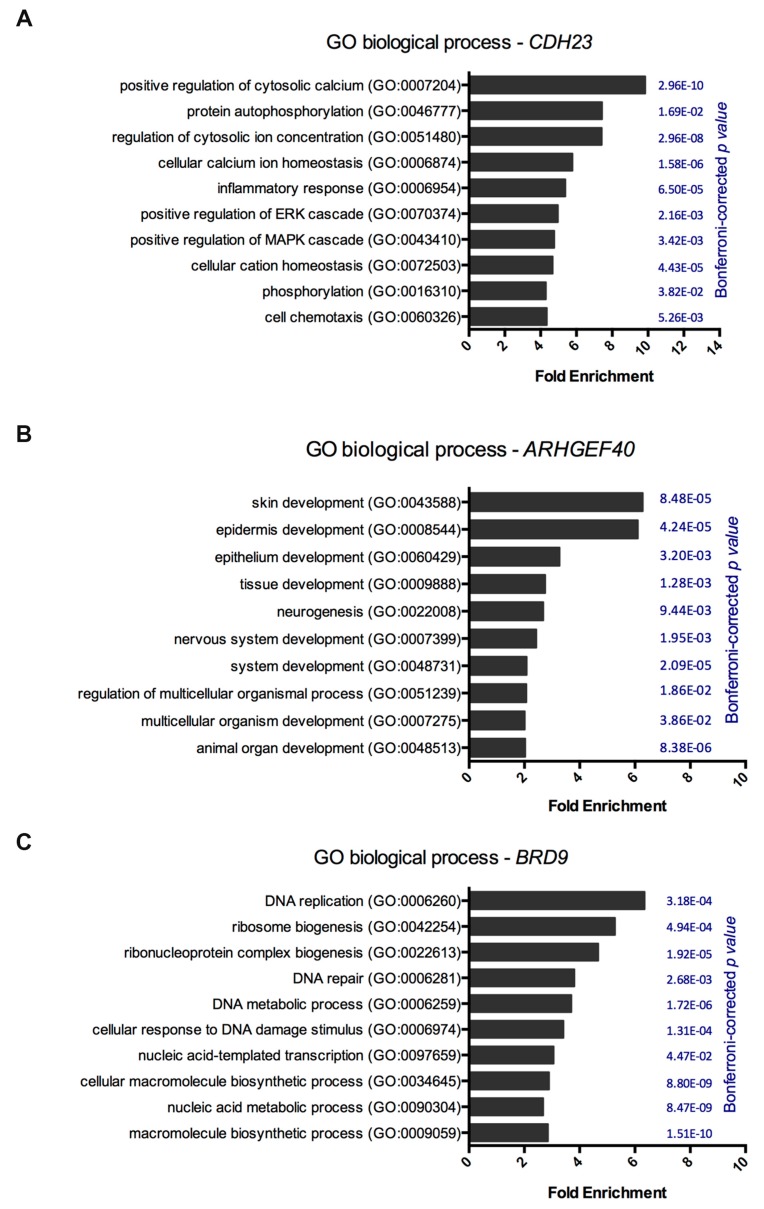
Gene ontology analysis of *CDH23*, *ARHGEF40,* and *BRD9* genes. Each gene expression was subjected to gene ontology and biological pathway enrichment analysis using PANTHER 14.0 against a *Homo sapiens* background reference. (**A**) Gene ontology and biological pathway enrichment for *CDH23* expression, (**B**) *ARHGEF40* expression, and (**C**) *BRD9* expression. Statistical over-representation was calculated using a binomial test and the results were considered significant at *p* < 0.05, after Bonferroni correction.

Notwithstanding, the expression of *CDH23*, *ARHGEF40,* and *BRD9* may be associated with patient prognostic in sporadic melanoma. To understand the biological relevance of the expression of these genes, Gene Ontology (GO) analyses were performed. Interestingly, *CDH23* revealed enrichment in pathways related to calcium/cation homeostasis, protein phosphorylation, inflammatory response, and positively regulating ERK and MAPK cascades (Figure 3A). Additionally, *ARHGEF40* gene showed significant enrichment in biological processes related to tissue development, mainly skin and epidermis (Figure 3B). The *BRD9* gene was enriched for DNA replication, DNA repair, and cellular response to DNA damage stimuli (Figure 3C). Altogether, these results suggested that *CDH23*, *ARHGEF40,* and *BRD9* genes could be also important for sporadic melanoma and consequently, a mutation in these genes could be pivotal in this disease.

**Figure 4:**
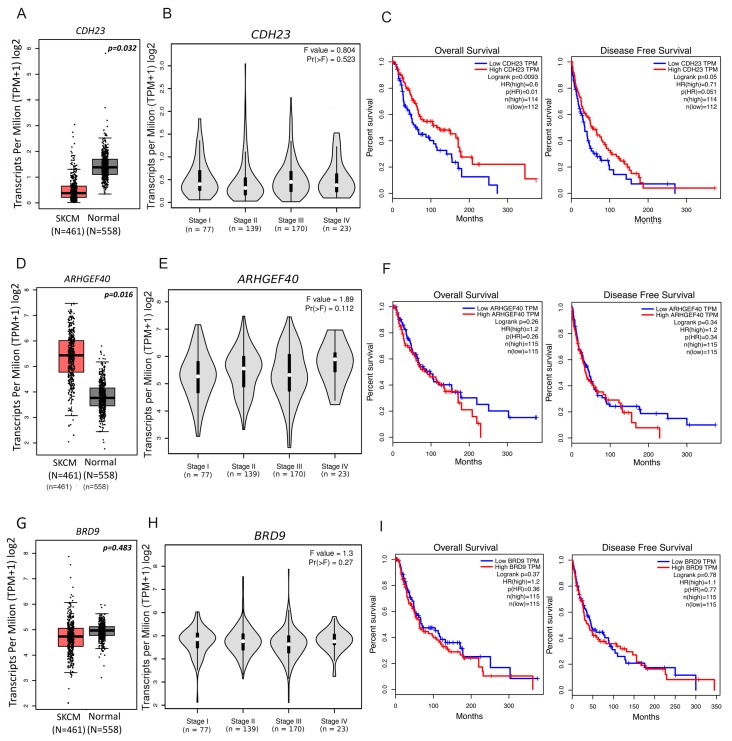
*CDH23*, *ARHGEF40,* and *BRD9* expression data from Gene Expression Profiling Interactive Analysis. Boxplots generated to compare gene expression levels between tumour and normal skin samples for (**A**) *CDH23*, (**D**) *ARHGEF40,* and (**G**) *BRD9*. Violin plots compare gene expression between tumour pathological stages for (**B**) *CDH23*, (**E**) *ARHGEF40,* and (**H**) *BRD9*. Expression data presented in log_2_ (transcripts per kilobase million + 1). One-way ANOVA was used for differential analysis. Overall survival and disease-free survival plots from high and low gene expressing tumour samples for (**C**) *CDH23*, (**F**) *ARHGEF40,* and (**I**) *BRD9*. Prognostic value was assessed using survival analysis from GEPIA program. Patients were divided in low and high expression groups based on median expression cut-off for each gene. Survival curves were analysed using the Cox PH model.

We then investigated each gene’s expression and its prognostic value in melanoma (Figure 4), revealing significant *CDH23* downregulation in cutaneous melanoma (SKCM) samples, when compared with normal skin samples (Figure 4A). Nevertheless, no relationship was found between gene expression and melanoma stage (Figure 4B). Interestingly, low *CDH23* expression significantly correlated with a worse overall (*p =* 0.0093; Figure 4C, left panel) and disease-free survival (*p* = 0.05; Figure 4C, right panel). Contrarily, *ARHGEF40* had a significantly higher expression in SKCM samples when compared to normal skin samples, although its expression did not appear to correlate with melanoma stages or either overall or disease-free survival (*p =* 0.26 and *p =* 0.34) (Figure 4D–F). No statistically significant associations were found between *BRD9* expression and tumour stage or survival (Figure 4G–I). This indicates that although *CDH23* is downregulated in melanoma and has a prognostic value (Figure 4C), it either has no correlation with melanoma aggressiveness or it possibly plays a role in early melanomagenesis.

## 4. Discussion

In the present study, we aimed to identify novel rare genetic variants responsible for hereditary melanoma susceptibility. Eight patients, with true features of hereditary melanoma such as MPM and high number of nevi which were negative for germline mutations in *CDKN2A*, *CDK4,* and *MITF* (p.E318K) genes were selected for WES. Interestingly, additional MPM and familial melanoma patients positive for *CDKN2A* or *MITF* (p.E318K) mutations did not harbour any of the suggestive variants identified in this study, supporting their probable relevant impact on MPM development.

Even though most variants identified in the MPM cases were polymorphisms, variants in the *CDH23*, *ARHGEF40,* and *BRD9* genes were rare in the databases employed, even among the healthy controls of the Portuguese population. One of the hardships of identifying hereditary melanoma is the fact that individuals with a history of familial melanoma may not actually have a genetic background in the family that makes them susceptible to melanoma. Rather, these families are subjected to similar environmental factors, such as profession and sun exposure, that lead to the development of melanoma. It is much more plausible that patients with true features of hereditary melanoma, such as MPM and high number of nevi, are carriers of a genetic background that culminates in melanoma susceptibility. Our promising variants were preferentially identified in patients with MPM. Therefore, it is possible that our patients with MPM have a hereditary background that makes them susceptible, and criteria for familial melanoma, which may be genetic and/or environmental background.

Since *CDH23*, *ARHGEF40,* and *BRD9* function is unclear, the impact of rare variants in these genes in melanoma context is unknown. Throughout our study, neither mutations on *CDH23*, *ARHGEF40,* and *BRD9* nor expression of *ARHGEF40* and *BRD9* affected the overall survival of the patients. However, the literature indicates that *CDKN2A*, one of the most important genes for melanoma susceptibility, also does not correlate with patient survival, despite its important association with melanoma susceptibility [35,36,37]. Hence, the three variants identified in the present study may play an important role in melanoma susceptibility.

For instance, *BRD9* has been identified as a subunit of the mammalian SWI/SNF chromatin remodelling complex [38] involved in organismal development, gene regulation, and cell lineage specification, which seems to be involved in tumour suppression [39]. *BRD9* is found overexpressed in numerous cancers and this overexpression seems to be associated with susceptibility to lung cancer, synovial sarcoma, and breast cancer [40,41,42], indicating that *BRD9* has a potential oncogenic effect. Moreover, a recent study revealed that the binding of *BRD9* to chromatin occurs at the enhancer level in a cell type-specific manner [43]. Additionally, *BRD9* chromatin-binding also regulates cancer cell proliferation and tumorigenicity in acute myeloid leukaemia, indicating its oncogenic role in transformed blood cells. This is in accordance with our results of Gene Ontology, which reveals that *BRD9* appears to be significantly associated with DNA replication and repair processes, particularly non-homologous end joining, implicated in cancer. However, despite this data from Gene Ontology analysis, no differential expression was observed between cutaneous melanoma and normal skin samples. Furthermore, no connection was found between *BRD9* expression and both overall and disease-free survival.

Although no statistically significant association between *BRD9* expression and tumour stage or survival was found in our study, *BRD9* importance must not be overlooked, since the available literature states that expression alterations in other important genes such as *CDKN2A* also do not correlate with patient prognosis in melanoma [35,36,37,44]; this could be the case of *BRD9*. Moreover, *BRD9* inhibition has been shown to result in decreased cell proliferation, G1-arrest, and apoptosis in rhabdoid tumour cell lines [45] and synovial sarcoma [41]. Overall, the data available in the literature regarding *BRD9* function coupled with our data from Gene Ontology analysis further highlights the importance of studying this gene in the context of melanoma.

As previously stated, *ARHGEF40*, or Solo, belongs to the Rho-guanine nucleotide exchange factor (Rho-GEF) family [32], having a role in maintaining cell and tissue integrity under mechanical stress [32]. Solo misfolding/aggregation may compromise the organization of F-actin and keratin fibres, and the localization of plakoglobin to cell-to-cell adhesion sites [46], the latter being particularly relevant in terms of tumorigenesis, since plakoglobin has been proposed to act as a tumour and metastasis suppressor. Several Rho-GEFs have been described as oncogenes, possibly due to deregulated activation of Rho GTPases [47]. Interestingly, according to our Gene Ontology data, *ARHGEF40* is particularly associated with skin and epidermal development, indicating that an *ARHGEF40* mutation could have a relevant impact on melanoma. Indeed, *ARHGEF40* has a significantly higher expression in cutaneous melanoma samples as compared to normal skin samples, although it has no significant relation with overall or disease-free survival. GEFs have also been associated with initiation and promotion of melanoma and basal cell carcinoma [48]. Therefore, *ARHGEF40* could also be associated with melanoma initiation. Additionally, *ARHGEF40* might regulate its protein activity through alternative splicing [32] and, according to our Human Splicing Finder tool results, the identified variant in this gene allowed the creation of an ESS site that blocks exon recognition. In turn, this blockade favours the silencing of splicing, and/or an alteration of an ESE site, which could disrupt splicing regulation [49]. Therefore, these splicing alterations could also have an important impact on ARHGEF40 function, consequently influencing the activation of Rho GTPases, which might result in numerous disorders, including cancer.

Similar to *ARHGEF40* and *BRD9,* the function of *CDH23* has not been established hitherto [47] in spite of being implicated in Usher syndrome type ID and non-syndromic hearing loss [50]. *CDH23* belongs to the cadherin family, a family that mediates calcium-dependent cell adhesion [51]. Here, *CDH23* was associated with biologic processes, such as the positive regulation of cytosolic calcium, regulation of cytosolic ion concentration, and cellular calcium ion homeostasis. As adhesion molecules, cadherins are known to participate in cancer metastasis, for instance E-cadherin and N-cadherin whose down or upregulation, respectively, can result in epithelial mesenchymal transition, a known marker for this event [52,53]. Furthermore, despite being reported that mutations in *CDH23* are commonly observed in hearing impairment conditions [51,52,53,54], they have also been associated with pituitary adenomas [55]. In fact, *CDH23* has been shown to be associated with a positive inflammatory response, which plays a pivotal role in cancer. Besides, as *CDH23* seems to regulate ERK and MAPK cascades, known melanoma-signalling pathways, mutations in this gene might play a role in MPM development by modulating the activity of these molecules.

In this study, we observed that cutaneous melanoma samples had a lower *CDH23* expression when compared to normal skin samples and this low expression was significantly correlated with a worse overall and disease-free survival. This suggests that a mutation causing downregulation or loss of function in this gene might be implicated in melanoma. The particular mutation herein identified introduces a possible site for mannosylation absent in wildtype CDH23, which is supported by the mounting evidence that aberrant patterns of cadherin glycosylation are linked to carcinogenesis and metastasis [56]. *CDH1*, also belonging to the cadherin family, encodes E-cadherin, which has been reported to have a tumor suppressor role [56,57], acting not only as an adhesive protein, but also as crucial in growth development and carcinogenesis. Besides the role of E-cadherin in metastasis and invasion [58], *CDH1* mutations were found in familial gastric cancer [59] and lobular breast cancer [60]. Additionally, its loss of expression has been reported in sporadic gastric cancer in distinct cohorts [61], and its downregulation was associated with poor outcome [62]. Overall, this data supports the hypothesis for the pathogenicity of this rare *CDH23* variant, as it could play a similar role to that of *CDH1* in gastric cancer.

Furthermore, our data reveals novel possibilities in the context of melanoma. Since *BRAF* and *NRAS* mutations are usually mutually exclusive [58] and clinical resistance to therapy involving these genes being a common issue in melanoma, finding new targets for treatment is crucial [60,61]. Since *CDH23* mutations may coexist with *BRAF* or *NRAS* mutations, in future studies it would be interesting to analyse if the presence of *CDH23* mutations could alter overall survival in *BRAF* or *NRAS* mutated melanoma patients, as it may lower patient overall survival and account for *BRAF* and *NRAS* resistance.

In summary, we have identified three novel mutations in *CDH23*, *ARHGEF40,* and *BRD9* genes, which could confer cutaneous melanoma susceptibility. Currently, despite our strong indications for importance of these genes, further studies are required to strengthen these findings. Our future studies will include an expansion of our cohort with several more cases for both MPM and familial melanoma. Nevertheless, the polymorphic variants identified showed a statistically significant cumulative effect on melanoma, demonstrating that they could contribute to increased MPM susceptibility in a polygenic manner. Besides, out of the three identified genes, *BRD9* seems to be the most promising in hereditary melanoma due to its involvement in oncogenic and DNA-repair mechanisms, which demonstrates the importance of studying this gene and its potential pathogenic variants in the future.

## Figures and Tables

**Table 1:** Candidate gene variants frequency. List of the frequency of candidate variants by screening against 26 MPM patients and 33 familial melanoma index cases, as well as their frequency in the Portuguese population by screening against 100 healthy controls.

Gene Name	Gene Alteration	MPM	Indexes	Healthy Controls
*NTN4*	c.1182C>T	(4/26) 15.4%	(0/37) 0.0%	(5/200) 2.5%
	p.Pro394 *			
*MTCL1*	c.4315G>A	(5/26) 19.2%	(2/37) 5.4%	(4/100) 4.0%
	p.Gly1439Ser			
*MAP2K3*	c.77G>C	(22/26) 84.6%	(33/37) 89.2%	(92/100) 92.0%
	p.Arg26Thr			
*CAND2*	c.992A>T	(3/26) 11.5%	(1/37) 2.7%	(3/100) 3.0%
	p.Glu331Val			
*RPL32*	c.98G>A	(3/26) 11.5%	(1/37) 2.7%	(2/100) 2.0%
	p.Arg33His			
*FNDC1*	c.3332G>A	(3/26) 11.5%	(1/37) 2.7%	(5/100) 5.0%
	p.Asp1112Asn			
*CDH23*	c.1096G>A	(2/26) 7.7%	(0/37) 0.0%	(2/300) 0.7%
	p.Ala366Thr			
CFAP47 *	c.4589A>C	(3/26) 11.5%	(2/37) 5.4%	(3/100) 3.0%
	p.His1530Pro			
BMX *	c.851C>T	(3/26) 11.5%	(1/37) 2.7%	(1/100) 1.0%
	p.Ser284Leu			
*ITIH3*	c.1130G>A	(4/26) 15.4%	(0/37) 0.0%	(2/100) 2.0%
	p.Arg377Gln			
*RNF213*	c.2122C>G	(4/26) 15.4%	(2/37) 5.4%	(3/100) 3.0%
	p.His708Asp			
*ARHGEF40*	c.2500C>T	(3/26) 11.5%	(0/37) 0.0%	(0/100) 0.0%
	p.Arg834Cys			
*BRD9*	c.183G>C	(4/26) 15.4%	(0/37) 0.0%	(0/100) 0.0%
	p.Glu61Asp			

* represents the gene variants that were excluded from the study for being identified as homozygous in a healthy control.

**Table 2:** Region-based aggregation tests for multiple variants. Region-based aggregation tests were performed to determine the cumulative effects of the multiple polymorphic variants identified in this study, namely, the burden test, the sequence kernel association test (SKAT) and the SKAT-O test.

Gene Alteration	MPM	Healthy Controls	Burden Test	SKAT	SKAT-O
*NTN4*	(4/26) 15.4%	(5/200) 2.5%	2.760892 × 10^−^^4^	9.48624 × 10^−^^4^	3.673208 × 10^−^^4^
**c.1182C>T**					
*MTCL1*	(5/26) 19.2%	(4/100) 4.0%			
**c.4315G>A**					
*CAND2*	(3/26) 11.5%	(3/100) 3.0%			
**c.992A>T**					
*RPL32*	(3/26) 11.5%	(2/100) 2.0%			
**c.98G>A**					
*FNDC1*	(3/26) 11.5%	(5/100) 5.0%			
**c.3332G>A**					
*ITIH3*	(4/26) 15.4%	(2/100) 2.0%			
**c.1130G>A**					
*RNF213*	(4/26) 15.4%	(3/100) 3.0%			
**c.2122C>G**					

## Supplementary Materials

The following are available online at https://www.mdpi.com/2073-4425/11/4/403/s1, Figure S1: Structural models of CDH23 p.Ala266Thr pathogenic variant, Figure S2: Structural models of ARHGEF40 (Solo) p.Arg834Cys pathogenic variant, Figure S3: Analysis of the frequency of mutation in additional datasets, Table S1: Selected variants after bioinformatics analysis and subsequent Sanger validation, Table S2: Variant impact prediction by *in silico* tools, Table S3: Structural models of CDH23 and ARHGEF40 obtained by homology modelling using the SWISS-MODEL server, Table S4: Primer list employed for variant validation, Table S5: Patient clinicopathological data.
